# Lokiarchaeota archaeon schizorhodopsin-2 (LaSzR2) is an inward proton pump displaying a characteristic feature of acid-induced spectral blue-shift

**DOI:** 10.1038/s41598-020-77936-9

**Published:** 2020-11-30

**Authors:** Keiichi Kojima, Susumu Yoshizawa, Masumi Hasegawa, Masaki Nakama, Marie Kurihara, Takashi Kikukawa, Yuki Sudo

**Affiliations:** 1 Graduate School of Medicine, Dentistry and Pharmaceutical Sciences, Okayama University, Okayama, 700-8530 Japan; 2 Atmosphere and Ocean Research Institute, The University of Tokyo, Chiba, 277-8564 Japan; 3 Faculty of Advanced Life Science, Hokkaido University, Sapporo, 060-0810 Japan; 4 Global Station for Soft Matter, GI-CoRE, Hokkaido University, Sapporo, 001-0021 Japan

**Keywords:** Bioenergetics, Ion transport, Membrane proteins

## Abstract

The photoreactive protein rhodopsin is widespread in microorganisms and has a variety of photobiological functions. Recently, a novel phylogenetically distinctive group named ‘schizorhodopsin (SzR)’ has been identified as an inward proton pump. We performed functional and spectroscopic studies on an uncharacterised schizorhodopsin from the phylum Lokiarchaeota archaeon. The protein, LaSzR2, having an all-*trans-*retinal chromophore, showed inward proton pump activity with an absorption maximum at 549 nm. The pH titration experiments revealed that the protonated Schiff base of the retinal chromophore (Lys188, p*K*_a_ = 12.3) is stabilised by the deprotonated counterion (presumably Asp184, p*K*_a_ = 3.7). The flash-photolysis experiments revealed the presence of two photointermediates, K and M. A proton was released and uptaken from bulk solution upon the formation and decay of the M intermediate. During the M-decay, the Schiff base was reprotonated by the proton from a proton donating residue (presumably Asp172). These properties were compared with other inward (SzRs and xenorhodopsins, XeRs) and outward proton pumps. Notably, LaSzR2 showed acid-induced spectral ‘blue-shift’ due to the protonation of the counterion, whereas outward proton pumps showed opposite shifts (red-shifts). Thus, we can distinguish between inward and outward proton pumps by the direction of the acid-induced spectral shift.

## Introduction

**Figure 1:**
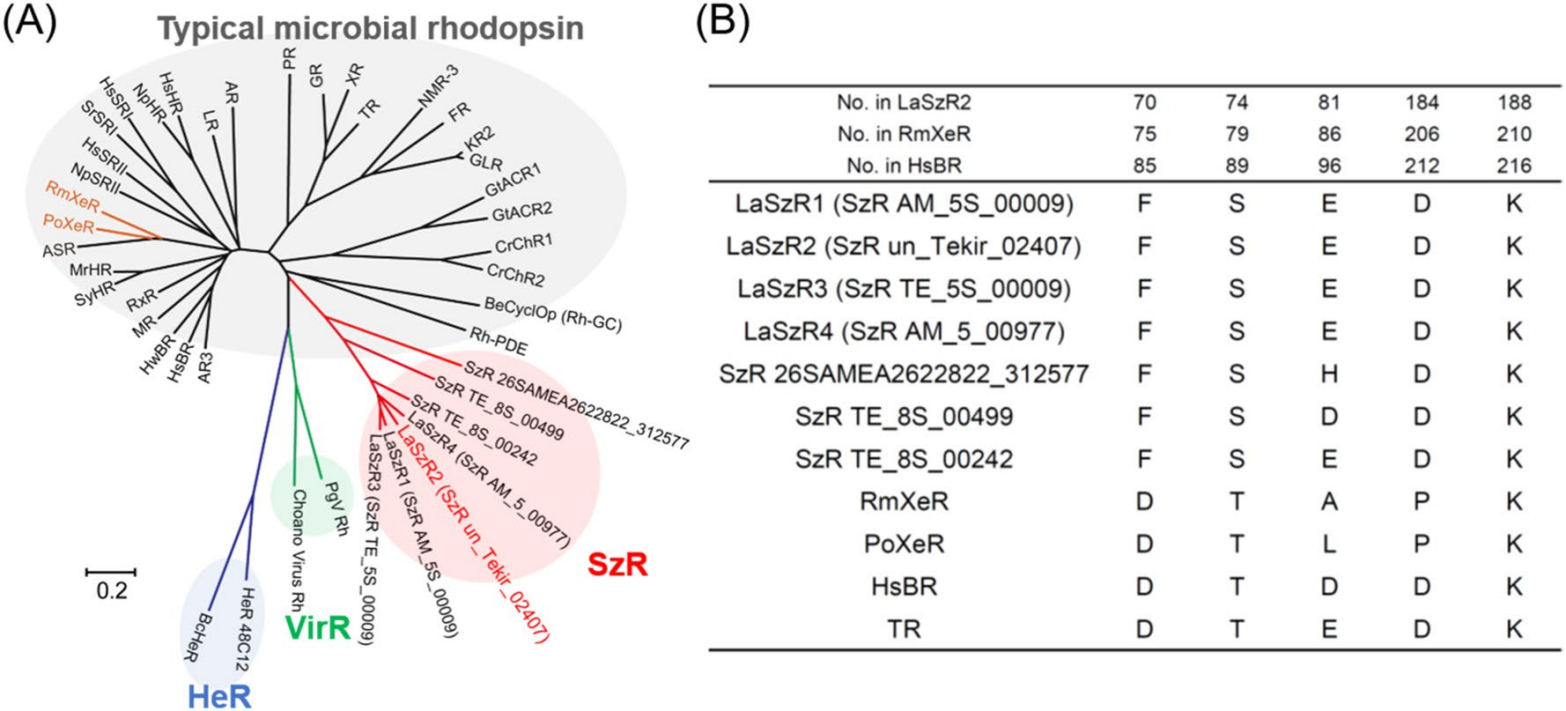
Introduction of schizorhodopsin (SzR). (**A**) The phylogenetic tree of typical microbial rhodopsins and newly discovered ones, heliorhodopsin (HeR), virus rhodopsin (VirR) and schizorhodopsin (SzR). In this study, we focused on one of the SzRs, LaSzR2 (red). The scale bar represents the number of substitutions per site. (**B**) The amino acid sequence alignment of SzRs and other microbial rhodopsins. The numbers of amino acids in LaSzR2, *Rm*XeR and *Hs*BR are indicated above the columns. The known functions of these amino acids are as follows: primary proton acceptor (Asp85), proton donor (Glu96), counterion (Asp212), Schiff base (Lys216).

Sunlight provides a fundamental source for organisms, including humans. Many organisms possess a variety of photoreceptor proteins that can capture sunlight. Among them, a seven-transmembrane protein, rhodopsin, has a retinal chromophore (vitamin-A aldehyde) inside the protein moiety and forms a huge phylogenetic cluster, it is widely distributed among archaea, bacteria, viruses and eukarya^1,2^. Recent elucidation of microbial genomic information has revealed novel phylogenetically distinctive clades containing various microbial rhodopsins, such as heliorhodopsin (HeR), virus rhodopsin (VirR) and schizorhodopsin (SzR) (Fig. 1A)^2–4^.

Historically, the archaeal proton pump rhodopsin was first discovered in 1971 from the extremely halophilic archaeon *Halobacterium salinarum* and named bacteriorhodopsin (*Hs*BR)^5^. *Hs*BR has been extensively studied from various aspects, and its ‘outward’ proton transport mechanism has been well-characterised at the atomic level^1,6,7^. Since 2000, eubacterial and eukaryotic proton pump rhodopsins, such as proteorhodopsin (PR), thermophilic rhodopsin (TR) and Leptosphaeria rhodopsin (LR), have been identified by genomic analysis and characterised, in addition to *Hs*BR^2,8^. These proton pumps convert light energy into available adenosine triphosphate (ATP) which provides chemical energy to the cells through the formation of a proton gradient across the cell membrane as a collaborative work with ATP synthase^9^, suggesting these ‘outward’ proton pumps play an important role in the ecosystem. In addition to these outward proton pumps, other ion pumps which transport different substrate ions such as Cl^−^ and Na^+^ have been identified in archaea, bacteria, viruses and eukarya (Fig. 1A, typical microbial rhodopsin)^2,8^. These ion pumps are thought to be involved in the maintenance of osmotic pressure in organisms^10^. Furthermore, light-gated cation and anion channels that act as photosensors responsible for phototaxis have been discovered mainly in algae (Fig. 1A)^2,8^. Another function of microbial rhodopsins is light-signal transduction^8^. In 1982, microbial rhodopsin, which was named sensory rhodopsin I (SRI), was discovered from the hyperhalophilic archaeon *H. salinarum* (Fig. 1A), and it has been shown to regulate both positive and negative phototaxis^11^. Then, in 1985, sensory rhodopsin II (SRII) was identified from the same organism as a similar photosensor only for negative phototaxis^12^. Light signals received by SRI and SRII are transmitted to a cytoplasmic signal transduction cascade, which regulates the direction of rotation of the flagellar motor, resulting in phototaxis responses^8,13^. Thus, *H. salinarum* cells are attracted to light with wavelengths longer than 520 nm for activation of *Hs*BR and avoid light shorter than 520 nm containing harmful near-ultraviolet (UV)^13^. In addition, enzymatic rhodopsins which have extended domains at the cytoplasmic side were also identified and characterised^8,13^.

Although the diversity of microbial rhodopsins has extensively expanded, it was believed that inward proton pumps do not exist in nature as they would hamper biological activities by impairing the production of the proton gradient. However, a phylogenetically distinctive rhodopsin clade, xenorhodopsin (XeR), has been identified from diverse eubacteria and archaea, such as *Parvularcula oceani*, *Rubricoccus marinus* and *Nanosalina* as a light-driven inward proton pump (Fig. 1A)^14–16^. These XeRs were named as *Parvularcula oceani* XeR (*Po*XeR), *Rubricoccus marinus* XeR (*Rm*XeR) and *Nanosalina* XeR (*Ns*XeR). Previously, a novel phylogenetically distinctive clade has been identified through metagenomic investigations and has been named schizorhodopsin (SzR) (Fig. 1A)^4^. Then, an SzR protein from Lokiarchaeota archaeon (SzR AM_5S_00009, Genbank accession number; TFG18381) was spectroscopically characterised by Inoue et al. as a light-driven inward proton pump^17^. In addition to the protein, several SzR proteins (SzR un_Tekir_02407, SzR TE_5S 00009 and SzR AM_5_00977, Genbank accession number; QBQ84358, TFG03937 and TFG21677) exist in the same origin; however, their spectroscopic properties have not been characterised. We rename these proteins as LaSzR1 (SzR AM_5S_00009)^17^, LaSzR2 (SzR un_Tekir_02407), LaSzR3 (SzR TE_5S 00009) and LaSzR4 (SzR AM_5_00977), respectively (Fig. S1). Identity and similarity of the protein sequence between LaSzR1 and LaSzR2 are 71% and 88%, respectively. These values are lower than those between SzR1 and SzR3 (85% and 95%), yet they are comparable with those between SzR1 and SzR4 (71% and 85%). In this study, we functionally and spectroscopically characterised LaSzR2. From the comparative study between LaSzR2, XeRs and outward proton pump rhodopsins such as BR and TR, we discuss a key characteristic of inward proton pumps, including LaSzR2.

## Results

### Light-induced inward H^+^ transport activity of LaSzR2

**Figure 2:**
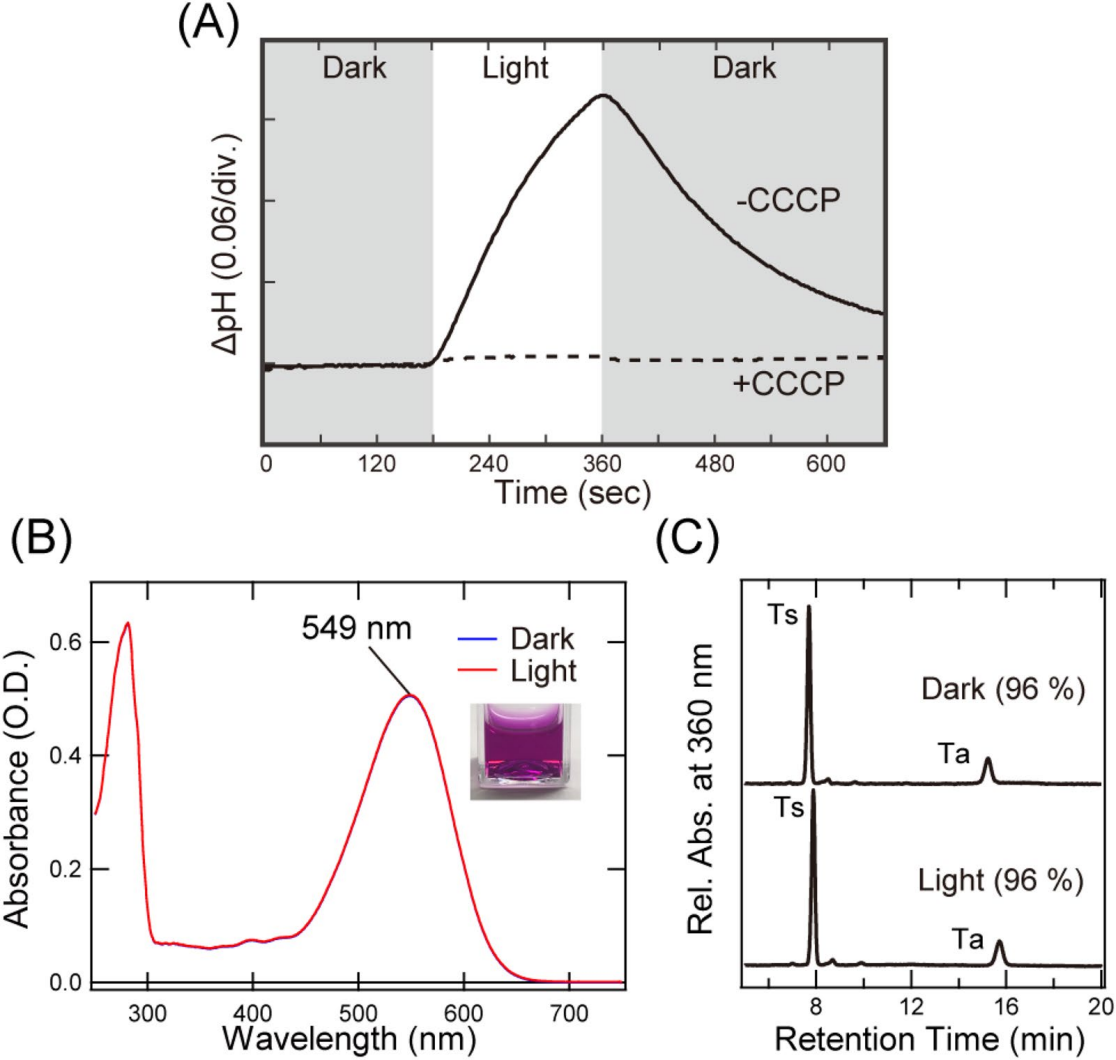
Function and absorption properties of LaSzR2. (**A**) Light-induced pH changes of *E. coli* cells expressing LaSzR2 in the absence (solid line) and presence (broken line) of 30 µM CCCP. The cell suspensions were placed under dark conditions (grey background) and illuminated at 520 nm for 3 min (white background). (**B**) The absorption spectra of the purified LaSzR2 in the dark (blue), and under light (red). The inset represents a photo of the purified LaSzR2 in the cuvette. (**C**) High-performance liquid chromatography (HPLC) patterns of LaSzR2 with and without green light irradiation for 10 min (550 nm). Ts and Ta represent all-*trans*-15-*syn* and all-*trans*-15-anti retinal oximes, respectively. The molar composition of each retinal isomer was calculated from the peak areas in the HPLC patterns and molar extinction coefficient of each isomer, as described previously^36^.

LaSzR2 was located in the SzR protein family on the phylogenetic tree, suggesting that LaSzR functions as a light-driven inward proton pump (Fig. 1A). The amino acids around the chromophore of LaSzR were well conserved with other SzRs (Figs. 1B and S1). Then, to investigate the function of LaSzR2, we measured its ion transport activity by monitoring light-induced pH changes of suspensions containing *E. coli* cells that expressed LaSzR2 as a recombinant protein. As shown in Fig. 2A, illumination of the *E. coli* suspension expressing LaSzR2 showed an increase in pH. The pH change was strongly impaired by the addition of the protonophore carbonyl cyanide m-chlorophenylhydrazone CCCP, indicating that LaSzR2 transports proton inwardly. Thus, we concluded that LaSzR2 functions as an inward light-driven proton pump, akin to LaSzR1^17^.

### The absorption spectrum and retinal composition of LaSzR2

Biological functions of microbial rhodopsins are tightly coupled with their spectroscopic properties. For instance, their absorption maxima are naturally optimised to absorb the cognate wavelengths of sunlight^9^. To quantitatively characterise the photochemical properties of LaSzR2, we employed the purified recombinant protein. Figure 2B shows the UV–Vis absorption spectra of LaSzR2 in both light- and dark-adapted forms; the absorption maxima are both located at 549 nm, indicating there is no light–dark adaptation in LaSzR2. The absorption at around 280 nm represents the absorption of aromatic residues of LaSzR2 such as Trp and Phe. From the ratio of absorptions at 280 nm and 549 nm, with the molecular coefficient of LaSzR2 at 549 nm (53,000 M^−1^ cm^−1^) (Fig. S2), we roughly estimated the purity of LaSzR2 as 73 %.

It is well known that rhodopsins have several retinal isomers such as all-*trans*, 13-*cis*, 11-*cis* and 9-*cis*^1^. For instance, *Hs*BR has both all-*trans* and 13-*cis* retinal chromophores at a 1:1 ratio in the dark-adapted form, while it becomes only all-*trans* (100%) in the light-adapted form^18^. Among inward proton pumps, *Rm*XeR and *Po*XeR have both all-*trans* and 13-*cis* forms as chromophores (45% and 55% for *Rm*XeR and 50% and 50% for *Po*XeR, respectively, in the light-adapted form)^14,15^. To confirm the retinal composition of LaSzR2, we performed high-performance liquid chromatography (HPLC) analysis of the light- and dark-adapted forms. Figure 2C shows the HPLC patterns of LaSzR2. In both forms, LaSzR is predominantly all-*trans-*retinal (96%), indicating no light–dark adaptation in LaSzR2. Thus, we succeeded in the purification of LaSzR2 with high purity and concluded that LaSzR2 functions with all-*trans-*retinal.

### p***K***_a_ values for the charged residues in LaSzR2

**Figure 3:**
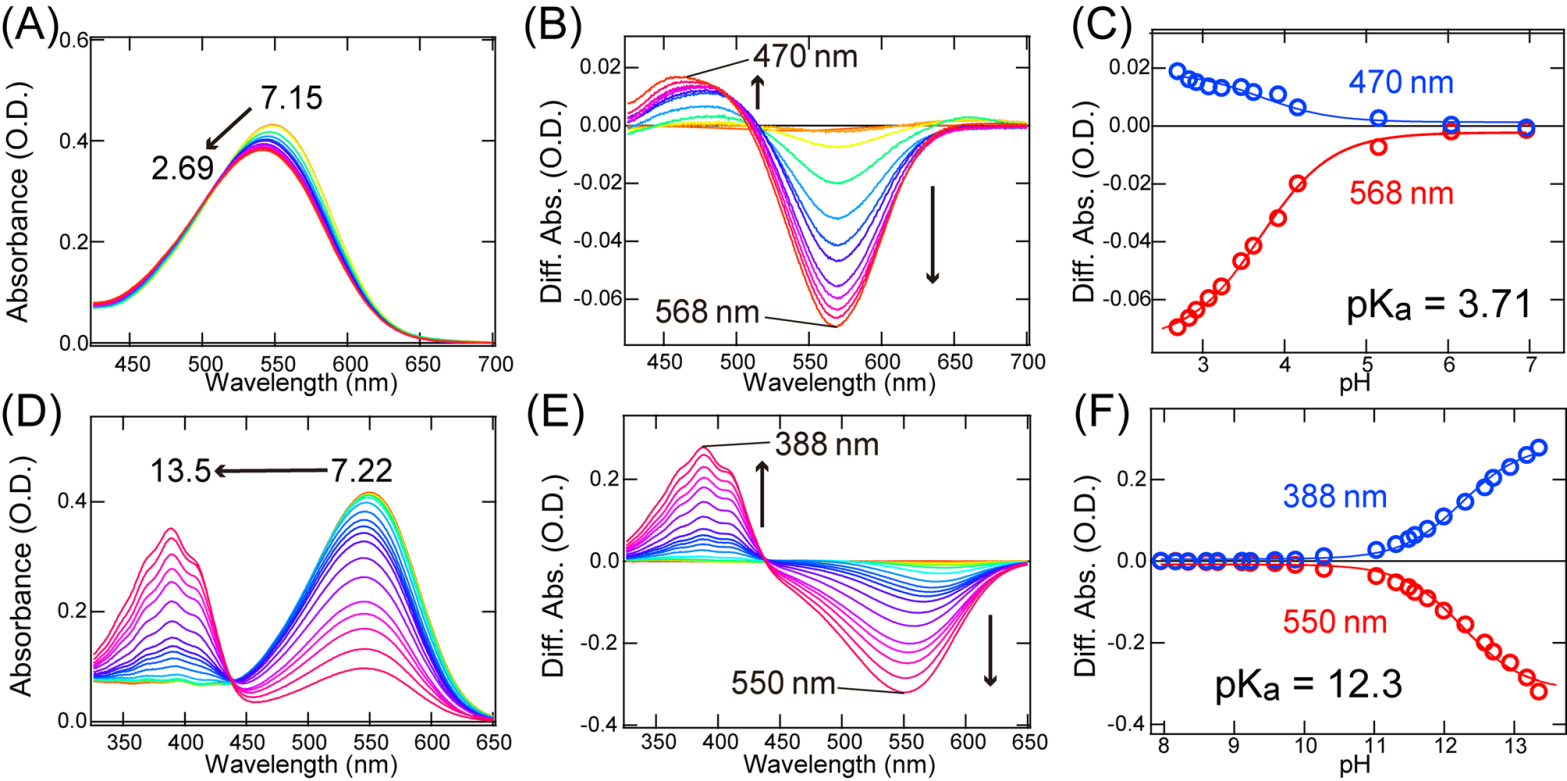
pH-induced spectral changes of LaSzR2. (**A**) The absorption spectra of LaSzR2 at acidic pH values (7.15–2.69). (**B**) The difference spectra at acidic pH values (6.96–2.69). The spectrum at pH 7.15 as the baseline was subtracted from each spectrum. (**C**) The pH-dependent absorption differences at 470 nm (blue open circles) and 568 nm (red open circles) were plotted against acidic pH values (6.96–2.69). The plots were fitted with the Henderson–Hasselbalch equation to estimate the p*K*_a_ value (solid red and blue lines). (**D**) The absorption spectra of LaSzR2 obtained at alkaline pH values (7.22–13.5). (**E**) The difference spectra at alkaline pH values (7.95–13.5). The spectrum at pH 7.22 as the baseline was subtracted from each spectrum. (**F**) The pH-dependent absorption differences at 388 nm (blue open circles) and 550 nm (red open circles) were plotted against alkaline pH values (7.95–13.5). The plots were fitted with the Henderson–Hasselbalch equation to estimate the p*K*_a_ value (solid red and blue lines).

It is well known that positively and negatively charged residues, such as Lys, Arg, Asp and Glu, play essential roles in the biomolecular functions of proteins, especially membrane-embedded ones. For instance, two carboxylates in the outward proton pump *Hs*BR (Asp85 and Asp212) form a salt-bridge with a specific Lys residue and are essential for the light-driven outward proton pump activity^19^. At the same time, a Na^+^ channel composed of a stator unit of the flagellar motor has a functionally essential Asp residue in the middle of the transmembrane domain^20^. We therefore estimated p*K*_a_ of charged residues for LaSzR2 by measuring its pH-dependent absorption spectra. The p*K*_a_ value for carboxylates involved in the colour change was first estimated as shown in Figs. 3A–C. Figure 3A shows absorption spectra at acidic pH values, where the absorption peak was blue-shifted from 549 to 541 nm. Figure 3B shows its difference spectra, where the spectrum at pH 7.15 as the baseline was subtracted from each spectrum. The difference spectra presented an isosbestic point at around 515 nm, indicating a direct transition from a deprotonated residue to a protonated one. The pH-dependent absorption at 470 nm and 568 nm were then plotted and were fitted with the Henderson–Hasselbalch equation assuming a single p*K*_a_ value (Fig. 3C). The p*K*_a_ value for carboxylates involved in the colour change could be estimated to be 3.71 ± 0.0375. It was confirmed that the spectral change is completely reversible, indicating is the equilibrium between the protonated and deprotonated forms of LaSzR2 at varying acidic pH. In general, a large spectral shift is due to the changes in the charge distribution around the retinal chromophore in rhodopsins. As Asp184 is only a carboxylate residue around the chromophore in the model structure of LaSzR2 (Fig. S3), we speculate that it shows the p*K*_a_ value and works as a counterion of the protonated Schiff base nitrogen. On the other hand, Glu81 is located in the intracellular side to the chromophore and is the second closest carboxylate to the Schiff base in the model structure of LaSzR2 (Fig. S3). It may be possible that Glu81 works as a counterion in LaSzR2 due to its reverse proton flow direction compared to outward proton pump rhodopsins. However, Glu81 is not conserved in SzRs while Asp184 is conserved in them (Fig. 1B), which suggests that Asp184 rather than Glu81 works as a counterion. To clarify this, we tried to express the D184N mutant in *E. coli* cells and purify it in detergent micelles by the same method for the wild-type. However, we did not obtain the purified mutant holoprotein due to less expression in *E. coli* cells or denaturation during the purification in the detergent. As a future work, further analyses such as X-ray crystallographic analysis and vibrational spectroscopy will be required to determine the counterion residue in LaSzR2.

We next estimated the p*K*_a_ value for the protonated Schiff base (Fig. 3D–F). Absorption maxima of microbial rhodopsins with the protonated Schiff base are generally known to appear at wavelengths above 450 nm. The fact that our spectrum of LaSzR2 presented the absorption maximum at 549 nm (Fig. 2B) indicates that the Schiff base was protonated at neutral pH. The deprotonation of the Schiff base nitrogen generally causes a spectral blue-shift to around 400 nm in all microbial rhodopsins^1^. Figure 3D shows absorption spectra at alkaline pH values, where the absorption increased at 388 nm and decreased at 549 nm. Figure 3E shows its difference spectra, where the spectrum at pH 7.22 as the baseline was subtracted from each spectrum. It presented an isosbestic point at around 440 nm, which imply a direct transition from the protonated Schiff base to the deprotonated one. Figure 3F plotted the absorption changes at 388 nm and 550 nm to perform the fitting using the Henderson–Hasselbalch equation assuming a single p*K*_a_ value. The p*K*_a_ value of the protonated Schiff base (Lys188) was then estimated to be 12.3 ± 0.0533. It was confirmed that the spectral change is completely reversible, indicating the equilibrium between the protonated and deprotonated Schiff base at varying alkaline pH.

### Photoreaction of LaSzR2

**Figure 4:**
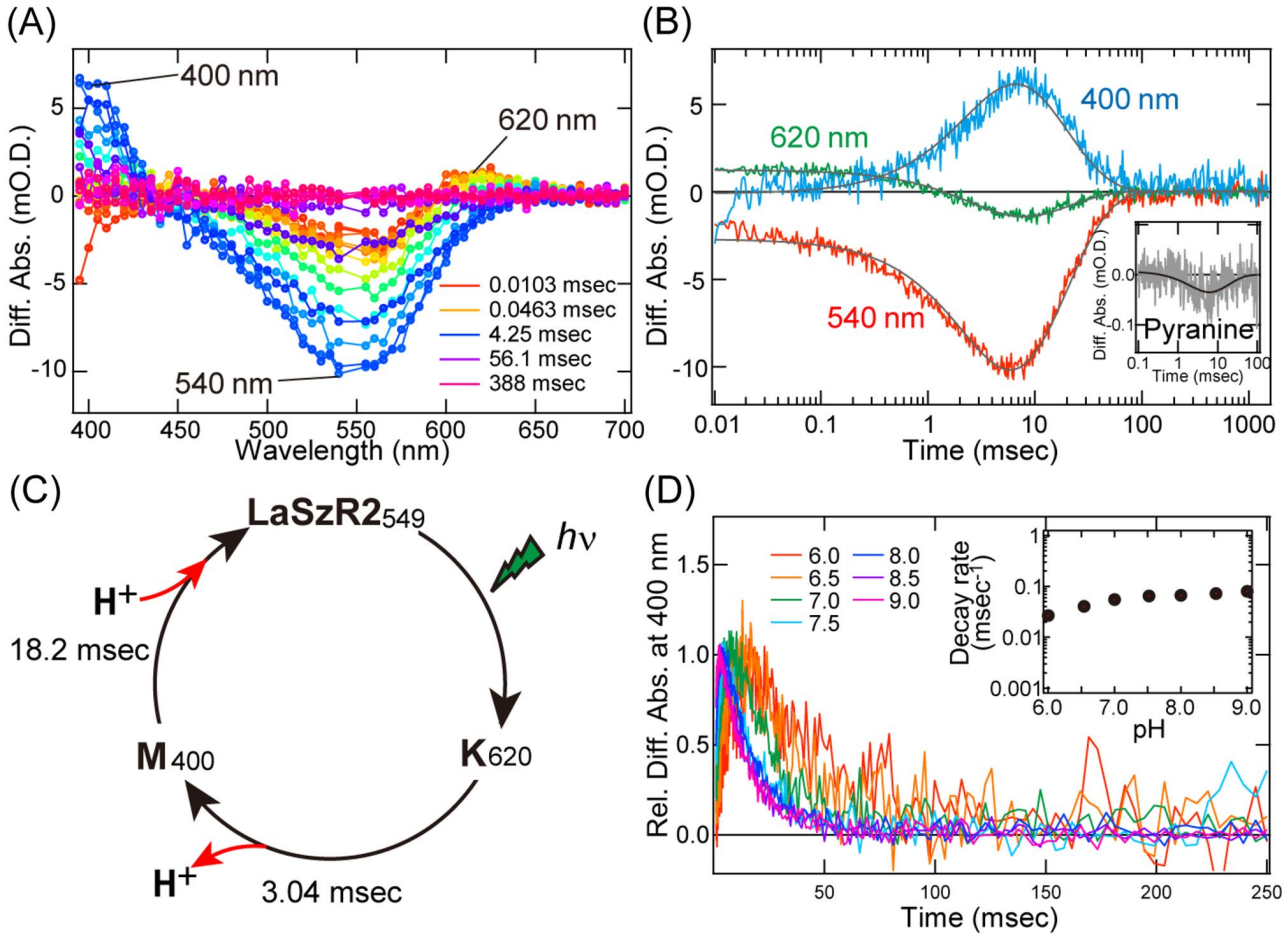
Photochemical reaction kinetics of LaSzR2 at 25 °C. (**A**) The flash-induced difference absorption spectra of LaSzR2 over a time range of 10.3 µs to 1.48 s. Depletion of the original state (540 nm) and simultaneous increases in the K (620 nm) and M (400 nm) intermediates were observed. (**B**) The flash-induced kinetic data at 620 nm, 400 nm and 540 nm representing the K-decay, M-decay and recovery of the original state, respectively. The curve for the K and M were well-fitted to a double exponential equation, and the decay time constants were estimated to be 3.04 and 18.2 ms, respectively. The inset indicates the difference in the absorption of pyranine in the presence of LaSzR2. (**C**) A putative photocycle model for LaSzR2 with the timing of proton release and uptake. (**D**) The M intermediate which was monitored at 400 nm at varying pH. The data were fitted by using a single exponential equation, and the decay rates were estimated (inset).

Microbial rhodopsins show the sequential photoreaction upon illumination, termed photocycle where the illumination triggers various photointermediates such as K, L, M, N and O, followed by it returning to the unphotolysed form^1^. During such the photocycle, microbial rhodopsins usually show the cognate biological functions, implying its biological importance. To investigate the photoreaction kinetics of LaSzR2, we carried out time-resolved flash-photolysis experiments by using the *E. coli* membrane that was expressing LaSzR2. Figure 4A shows the flash-induced difference spectra over the spectral range of 380–700 nm and the temporal range of 10.3 µs–1.48 s. Upon the excitation of the original state, a negative peak appeared at around 540 nm within 10 µs and recovered within 100 ms (Fig. 4A,B), which can be interpreted as the depression and recovery of the original state. Meanwhile, a positive peak also appeared at around 620 nm within 10 µs (Fig. 4A,B). The formation of red-shifted K intermediate was observed within ~ 10 µs in several microbial rhodopsins, such as PR and a sodium pump rhodopsin, KR2^21,22^. Given the occurrence time-scale and the absorption maximum, we can tentatively assign the peak at around 620 nm to the K intermediate (LaSzR2_K_). Next, the decay of the K intermediate and the concomitant formation of the blue-shifted intermediate were observed at around 400 nm in the order of milliseconds (Fig. 4A,B). This typical absorption change allows us to tentatively assign it to the emergence of the M intermediate (LaSzR2_M_). Finally, the decay of the M intermediate and the concomitant recovery of the original state were observed at around 540 nm as the final step in a series of the photoreactions. Figure 4B shows the time courses of the absorbance changes at three representative wavelengths (540 nm, 620 nm and 400 nm for the original state, K and M intermediates, respectively). The decay rates for the K and M intermediates were respectively estimated as 3.04 ms and 18.2 ms by the fitting with an exponential function. We then investigated when H^+^ uptake and release happen during the photocycle using the pH-sensitive dye pyranine as a pH indicator. Since the absorption change of the pyranine corresponds to the pH change of the bulk solution^23^, it allows us to monitor H^+^ uptake or release of rhodopsins upon illumination. The inset of Fig. 4B shows the absorption changes of the pyranine during the photocycle in the presence of LaSzR2. The initial decrease (τ = 2.58 ms) and subsequent increase (τ = 17.6 ms) of the absorption indicate that H^+^ is initially released and is subsequently taken up from the bulk. Notably, the absorption changes of pyranine roughly matched with that seen in the formation and the decay of the M intermediate (Fig. 4B) which suggests that H^+^ release and uptake happen concurrently with the formation and decay of the M intermediate. Taken these results together, we here speculate the photocycle model of LaSzR2 with the timing of H^+^ release and uptake (Fig. 4C). Upon the excitation of the original state, the red-shifted K intermediate and the blue-shifted M intermediate appear sequentially and then return to the original state.

In the outward proton pumps (*Hs*BR and AR3), the decay of the M intermediate corresponds to the proton movement from the conserved proton donating residue (Asp96 in *Hs*BR) to the deprotonated Schiff base nitrogen (Lys216 in *Hs*BR)^1,19^. This internal proton movement is one of the rate-limiting steps in microbial rhodopsins, indicating the importance of rhodopsin function. Moreover, since this reaction occurs in the internal protein moiety, the M-decay is generally insensitive to the bulk pH^24^. On the other hand, for *Natronomonas pharaonis* sensory rhodopsin (*Np*SRII), this residue is replaced by the neutral residue Phe86, resulting in the formation of a long-lived M intermediate (with a life-time of a few seconds). The slow photocycle allows the transient accumulation of signalling states of the receptors to catalyse a sustained phosphorylation cascade that controls flagellar motor rotation^8,13^. Moreover, since the proton directly came from the bulk environment in this instance, the M-decay is highly sensitive against the bulk pH^25^. In addition, the F86D mutant of *Np*SRII showed a three-fold faster decay of the M intermediate when compared with the wild-type^26^. To investigate the presence of a proton donating residue, we measured LaSzR2_M_ decay at varying environmental pH levels. As seen in Fig. 4D, the decay traces of LaSzR2_M_ was very similar across the entire pH scale. The curves fitted well to a single exponential equation, and the decay rate constants were plotted against the environmental pH as logarithmic functions (Fig. 4D, inset). The pH-insensitivity of LaSzR2_M_ suggests that the deprotonated Schiff base can be protonated by a proton from the internal amino acid. In addition, the M-formation seems to be pH-insensitive (Fig. 4D), suggesting that this step is also composed of no proton transport either from or to the bulk environment.

## Discussion

**Figure 5:**
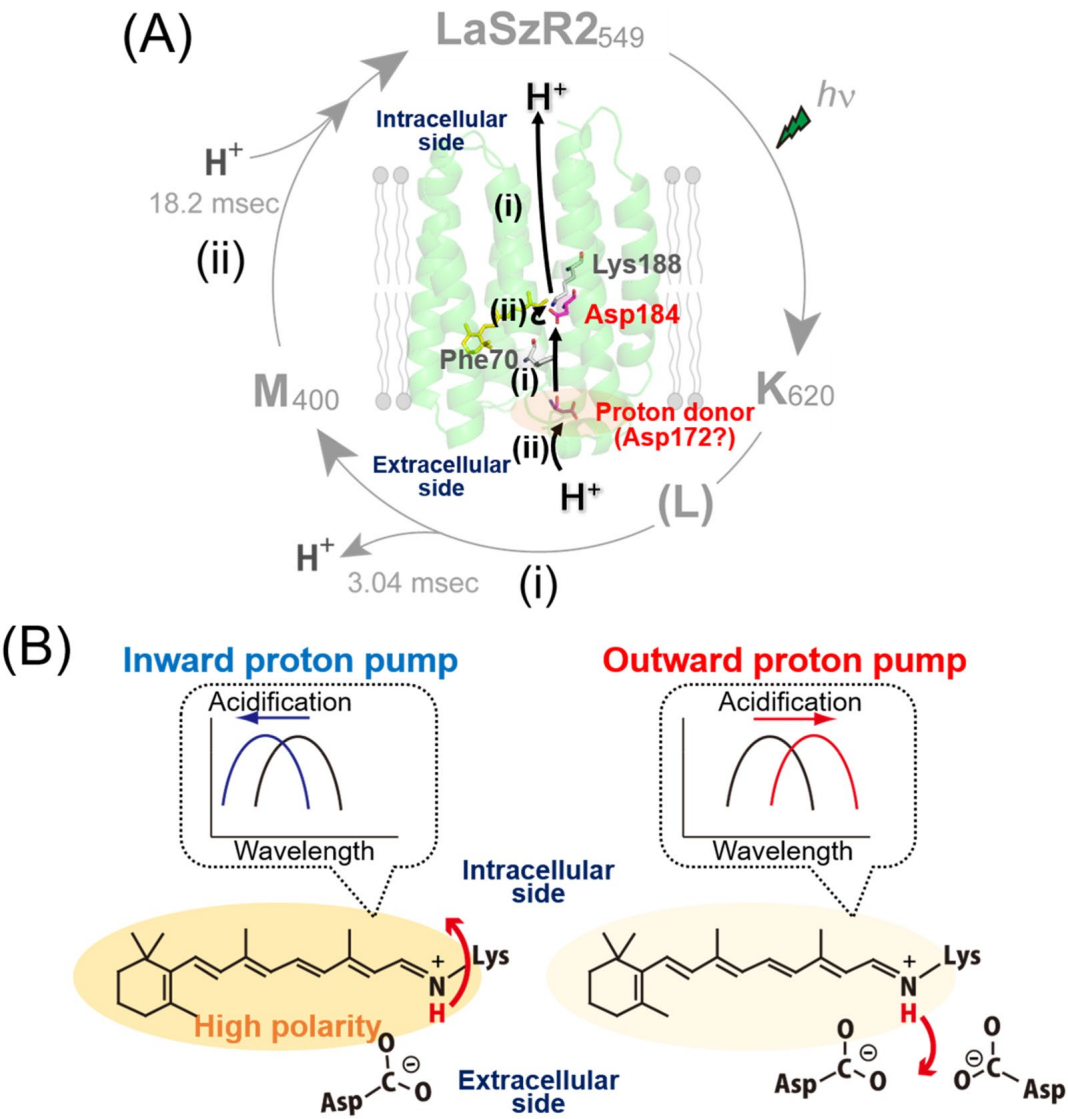
A hypothetical model for proton transport mechanism in LaSzR2. (**A**) An inward proton transport mechanism in LaSzR2 during the photocycle. The pathway for putative proton transport and key residues are indicated on the homology model of LaSzR2, which was constructed from the crystal structure of *Nanosalina* XeR (PDB 6EYU) by SWISS model (https://swissmodel.expasy.org/). (**B**) A key difference between inward and outward proton pump rhodopsins. Inward proton pump rhodopsins show acid-induced spectral blue-shifts while outward proton pump rhodopsins show acid-induced spectral red-shifts. We speculate that the high polarity of the retinal chromophore in inward proton pumps is a key determinant of the direction of proton movement.

From the results and other findings, we speculate a hypothetical model for an inward proton transport mechanism in LaSzR2 (Fig. 5A). In outward proton pump rhodopsins, including *Hs*BR and PR, a conserved carboxylate (Asp85 in *Hs*BR) works as a primary counterion of the Schiff base. There is also another carboxylate (Asp212 in *Hs*BR) which acts as a secondary counterion (Figs. 1B and S1)^1^. In XeRs, the primary counterion is conserved at the same position with *Hs*BR as an Asp residue (D74 in *Rm*XeR), whereas the secondary one is replaced by the neutral Pro residue (P206 in *Rm*XeR) (Figs. 1B and S1). Our mutational studies on the carboxylates of *Rm*XeR revealed that D74 had been assigned as the counterion (Fig. 1B and Table 1)^15^. Whereas in SzRs, only the secondary counterion for *Hs*BR is conserved as a carboxylate (Asp184 in LaSzR2) (Fig. 1B). Since no other carboxylate is located around the Schiff base of the retinal in the model structure of LaSzR2 (Fig. S3), we tentatively assigned a primary counterion for LaSzR2 to Asp184. Thus, together with the results of the pH titration experiments, we assumed that at a neutral pH, the Schiff base nitrogen that has a p*K*_a_ value of 12.3 (Lys188) in LaSzR2 is protonated and interacts with its deprotonated Asp184, which has a p*K*_a_ value of 3.7 (Fig. 5A). Upon illumination, the all-*trans-*retinal chromophore in LaSzR2 is presumably isomerized to the 13-*cis* form with a change of direction of the N–H group, leading to the production of two spectroscopically distinct intermediates, K and M, during the photocycle (Fig. 5A). The scheme is substantially similar to those of other inward and outward proton pumps^1,14,15^. The previous study suggested that the blue-sifted L intermediate appeared after the formation of the K intermediate and then decayed into an M intermediate in two SzRs (SzR AM_5S_00009 and SzR 26SAMEA2622822_312577)^17^. LaSzR2 did not show an absorbance change of the L intermediate within the time resolution of our measurements. Therefore, it can be postulated that the accumulation of the L intermediate is low in the photocycle of LaSzR2, presumably due to the fast L-decay.

**Table 1:** The molecular properties of microbial rhodopsins.

Opsin	Origin	All-*trans*-retinal	p*K*_a_ of the counterion	Acid-induced spectral shift	M-decay (ms)	Proton donor	Refs.
**Inward proton pump**							
*SzR*							
LaSzR2 (SzR un_Tekir_02407)	Archaea	96% (Dark)	3.7 (Asp 184?)	Blue (Δ = 8 nm)	18.2	Yes (Asp 172?)	This study
		96% (Light)					
LaSzR1 (SzR AM_5S_00009)	Archaea	94% (Dark)	N.D.	N.D.	25.5 and 60	N.D. (No?)	^17^
		73% (Light)					
SzR 26SAMEA2622822_312577	Archaea	N.D.	N.D.	N.D.	2.68 and 140	N.D.	^17^
*XeR*							
*Rm*XeR	Bacteria	75% (Dark)	2.1 (Asp 75)	Blue (Δ = 34 nm)	100	No?	^15^
		45% (Light)					
*Po*XeR	Bacteria	92% (Dark)	N.D.	N.D.	203	Yes (unidentified)	^14^
		50% (Light)					
ASR	Bacteria	67% (Dark)	4.0 (Asp75)	Blue (Δ = 3 nm)	50–60	N.D.	^30,41^
		20% (Light)					
**Outward proton pump**							
*Hs*BR	Archaea	47% (Dark)	2.7 (Asp85)	Red (Δ = 36 nm)	0.5	Yes (Asp96)	^18,27^
		100% (Light)					
TR	Bacteria	98% (Dark)	3.4 (Asp95)	Red (Δ = 15 nm)	260	N.D.	^28^
		96% (Light)					
ARI	Eukarya	94% (Dark)	2.7 (Asp89)	Red (Δ = 22 nm)	38 and 313	Yes (Asp100)	^29,42^
		98% (Light)					

The putative proton translocation model for LaSzR2 is as follows: (1) changes in the pyranine signals indicated that the H^+^ is released to the intracellular bulk environment upon the formation of the M intermediate. Judging from the large spectral blue-shift to around 400 nm upon M-formation, the Schiff base should be deprotonated in the M intermediate as well as other microbial rhodopsins including *Hs*BR and *Rm*XeR. In general, for outward proton pump rhodopsins, the proton of the Schiff base nitrogen is transferred to its counterion upon the formation of the M intermediate^1^. However, the proton movement should be from the extracellular side to the intracellular side for LaSzR2, because of its inward proton transport phenomenon. Therefore, we tentatively speculate that the released proton upon M intermediate formation is from the Schiff base nitrogen (Fig. 5A). In this case, charge balance around the retinal chromophore is disrupted upon deprotonation of the Schiff base. Therefore we assumed that the putative counterion Asp184 is protonated by a proton from somewhere else. As described, the formation and decay of the M intermediate are generally pH-insensitive, suggesting the presence of a proton donating residue in the protein moiety. From the view-point, Asp172 would be the first candidate for the residue as it is the only carboxylate around the extracellular side. Thus, in addition to the proton release from the Schiff base, a proton of Asp172 would be transferred to the primary counterion Asp184 (Fig. 5A). (2) Then, during the M-decay, the Schiff base is reprotonated by the proton movement from Asp184. Simultaneously, Asp172 is also reprotonated by the proton from the extracellular bulk environment; this is experimentally supported by the changes in the pyranine signal (Fig. 4B). During the recovery of the original state, the retinal chromophore returns to the all-*trans-*configuration from analogy with other microbial rhodopsins^1^. Thus, one proton is transported from the extracellular side to the intracellular side during a photocycle (Fig. 5A). To clarify the hypothetical model for an inward proton transport mechanism in LaSzR2, the structural and mutational analysis will be necessary in a future work.

The functional and photochemical properties of LaSzR2 are listed in Table 1, as well as other microbial rhodopsins, other SzRs, XeRs and other outward proton pumps (*Hs*BR, TR and ARI). From the similarity and dissimilarity between inward and outward proton pumps, we would like to extract the key differences between them. (1) First, some of the proton pumps, LaSzR1, XeRs and *Hs*BR, showed light–dark adaptation of the retinal chromophore, such as changes in the retinal configuration upon illumination, while no adaptation was observed for LaSzR2, TR and ARI. Thus, isomer composition of the retinal chromophore in the inward and outward proton pumps is not related to their function. (2) Second, all rhodopsins have deprotonated counterion at neutral pH, and the p*K*_a_ ranges from 2.1 to 4.0 with no correlation between the direction of proton transport in the rhodopsins (Table 1). (3) Third, we focused on the acid-induced spectral shift. It has been reported that for most microbial rhodopsins, including outward H^+^ pumps such as *Hs*BR, AR3, TR and ARI, a broad spectral red-shift is observed under acidic pH conditions (approx. 20–30 nm). The residue showing the p*K*_a_ value is assigned to be a conserved Asp residue (Asp85, Asp95 and Asp 89 for *Hs*BR, TR and ARI, respectively)^27–29^. In contrast, we previously showed that an inward proton pump *Rm*XeR shows a large spectral blue-shift^15^ while it remains unknown whether *Po*XeR shows acid-induced blue-shift^14^. It has been reported that microbial rhodopsin belonging to the XeR clade, *Anabaena* sensory rhodopsin (ASR) functions as a light-dependent transcription regulator and shows a light-induced inward H^+^ movement as well as other XeRs. For ASR, a slight acid-induced spectral blue-shift has been reported (4 nm)^30^. During the revision process of our paper, Harris et al. reported that a new member of SzRs (AntR) shows a light-induced inward H^+^ transport with acid-induced blue-shift as like LaSzR2^31^ while it remains unknown whether LaSzR1 shows acid-induced blue-shift^17^. Thus, the direction of acid-induced spectral shift seems to be tightly correlated with the direction of proton transport in inward and outward proton pumps (Table 1). (4) Fourth, the decay rate of the rate-limiting step during the photocycle (M-decay) ranges from 0.5 to 313 ms (Table 1) for inward and outward proton pumps, with no correlation between the direction of proton transport. (5) Fifth, LaSzR2 seems to have a proton donating residue (presumably Asp172) akin to *Hs*BR and ARI, however, the presence or absence of the proton donating residue does not correlate with the direction of proton transport. Thus, comparison of the photochemical properties of LaSzR2 and other proton pump rhodopsins revealed that we should distinguish the direction of the proton pump by direction of the acid-induced spectral shift. To verify the hypothesis, the comprehensive confirmation of the acid-induced blue-shift for various inward proton pump rhodopsins will be necessary in future works.

Retinylidene proteins are capable of tuning their absorption spectrum extensively depending on their interaction between the opsin (apoprotein) and the retinyl chromophore, termed the opsin-shift^32^. Such the opsin-shift relies on the difference in the electronic energy gap between ground- and excited states of the retinal chromophore^32^. Several mechanisms for the shift have been proposed based on previous experimental and theoretical works: (1) Electrostatic interaction between the retinal and the negatively charged carboxylate, that is, counter ion. (2) An alteration in polarizability or polarity of the environment of retinal binding pockets due to the arrangement of residues lining the binding pocket. (3) Twisting the 6-S bond that connects the polyene chain to the β-ionone ring as well as the distortion of the retinal due to steric interactions with residues lining the binding pocket. These effects would be the explanation that the retinylidene proteins exhibit extensively variable absorption maxima in the visible light region (360–620 nm). In general, as aforementioned, microbial rhodopsins showed acid-induced spectral red-shifts by protonation of the counterion residue (Asp85 in *Hs*BR). This is explained by the changes in the interaction between the retinal chromophore and its counterion. One of the exceptions is the inward chloride pump, *H. salinarum* halorhodopsin (*Hs*HR), in which the chloride-binding induced a spectral red-shift (~ 10 nm)^33,34^. The X-ray structure of *Hs*HR revealed that the chloride (Cl^−^) binds to the same position of the counterion of outward proton pumps such as Asp85 in *Hs*BR^34^. Therefore, chloride works as a counterion of the protonated Schiff base, and its binding leads to the spectral red-shift. This abnormal spectral shift is theoretically solved as changes in the electronic polarisation of the protein environment^35^. In the same way, we speculate that the retinal chromophore of inward proton pumps, such as LaSzR2 and *Rm*XeR, is highly polarised in comparison with outward proton pumps, which is a key determinant of the direction of proton movement (Fig. 5B).

## Conclusion

The photoreactive protein rhodopsin is widely distributed among various organisms and viruses and has diverse biological functions. In this study, we characterised the function and photochemical properties of a newly identified schizorhodopsin, LaSzR2. From the results, we speculated its inward proton transport mechanism. In addition, a comparison of the properties of LaSzR2 and other proton pumps revealed that we could distinguish between inward and outward proton pumps by the direction of acid-induced spectral shifts.

## Methods

### Gene preparation, sequence alignment and phylogenetic analysis

The gene and protein sequences of LaSzR2 was obtained from the Genbank database (Accession number; QBQ84358)^4^. The protein sequences for schizorhodopsin (SzRs) and other microbial rhodopsins, *Rm*XeR (WP_094549673), *Po*XeR (WP_051881467), *Hs*BR (CAP14056) and TR (WP_014629850), were aligned by ClustalW. A gene for LaSzR2 was chemically synthesised by Eurofins Genomics (Tokyo, Japan) with NdeI and XhoI restriction enzyme sites at each terminus; the codon was optimised for *Escherichia coli*. The *E. coli* strain DH5α was used as a host for DNA manipulation. The DNA fragment containing LaSzR2 was inserted into the pET21a(+) vector using the NdeI and XhoI restriction enzymes. Finally, the plasmid encoded for hexahistidine at the C-terminus, which was utilised for purification of the expressed protein. D184N mutant gene was constructed using the In-Fusion Cloning Kit (Takara Bio, Inc., Japan) according to the manufacturer’s instructions^36^. The phylogenetic tree was inferred using the maximum likelihood method of the MEGA X software (https://www.megasoftware.net/).

### Ion transport measurements

The *E. coli* strain C41 (DE3) (Lucigen, Middleton, WI, USA) was used as a host for ion transport measurements, as described previously^37^. The *E. coli* cells harbouring the plasmid were grown in 2 × YT medium supplemented with ampicillin (final concentration = 100 µg/mL) until an optical density at 660 nm (OD_660_) reached 0.3–0.6. Then, the cells were incubated at 37 °C for 3–4 h by adding 0.1 mM isopropyl β-D-1-thiogalactopyranoside (IPTG) and 10 µM all-*trans*-retinal (Sigma-Aldrich, St. Louis, MO, USA) for protein expression induction. The LaSzR2-expressing cells were collected by centrifugation (4400×*g* for 3 min) and washed more than three times in 100 mM NaCl and resuspended in 6 mL of the same solution for measurements. The cell suspension was then placed in the dark for several minutes and illuminated for 3 min using a 300 W xenon lamp (MAX-303, Asahi spectra Co. Ltd., Japan) with a band-pass filter of 520 ± 10 nm (MX0520, Asahi Spectra Co. Ltd.). Measurements were repeated under the same conditions after the addition of the protonophore carbonyl cyanide m-chlorophenylhydrazone (CCCP) (final concentration = 30 µM). Light-induced pH changes were monitored using a pH electrode (LAQUA F-72 pH metre, HORIBA, Ltd., Kyoto, Japan). All measurements were kept at 5 °C using a thermostat.

### Protein purification

For protein purification, the *E. coli* strain BL21 (DE3) was used as a host, as described previously^15,38^. The *E. coli* cells harbouring the plasmid were grown at 37 °C in 2 × YT medium supplemented with ampicillin (final concentration = 50 µg/mL). The protein expression was induced at OD_660_ of 1.4–1.6 with 1 mM IPTG and 10 μM all-*trans*-retinal. Then, the cells were disrupted by sonication for 120 min on ice-cold water in a buffer containing 50 mM Tris–HCl and 300 mM NaCl (pH 7.0). The crude membrane fraction was collected by ultracentrifugation and solubilised with 1.0 w/v % *n*-dodecyl-β-d-maltoside (DDM). The solubilised-fraction was purified by Ni^2+^ affinity column chromatography with a linear gradient of imidazole, as described previously^15,38^. The purified protein was concentrated by centrifugation using an Amicon Ultra filter. The sample media was then replaced by the appropriate buffer solution by ultrafiltration more than three times.

### Spectroscopic measurements

UV–visible spectra were recorded using a UV-2450 and UV-2600 spectrophotometer (Shimadzu Co., Kyoto, Japan) at room temperature (approx. 25 °C) in the appropriate buffer solution. The molar extinction coefficient of LaSzR2 was determined by monitoring the reaction of LaSzR2 with hydroxylamine (final concentration of 100 mM) as described previously^39^, where the molar extinction coefficient of retinal oxime (33,600 M^−1^ cm^−1^) was utilized. The retinal compositions were analysed by HPLC, as described previously^3,15^. For light-adaptation, the samples were illuminated for 10 min at 550 ± 10 nm for LaSzR2, where the light power was adjusted to approximately 5 mW/cm^2^. This was enough power to induce light-adaptation of archaerhodopsin-3 (AR3), as reported previously^40^. The molar compositions of the retinal isomers were calculated from the areas of the peaks in the HPLC patterns that were monitored at 360 nm, as described previously^3,15,36^.

For pH titration experiments, the purified samples were suspended in a seven-mix buffer (Tris, citric acid, MES, HEPES, MOPS, CHES and CAPS, 10 mM each) containing 300 mM NaCl and 0.05 w/v % DDM. The absorbance of the sample was approx. 0.4 O.D. at 549 nm (approx. 8 μM). The pH was adjusted to the desired value by adding concentrated HCl or NaOH; the absorption spectra of the samples were measured at each pH. All measurements were conducted at room temperature (approx. 25 °C). For data analysis, the absorption differences (*ΔAbs*) at specific wavelengths were plotted against pH, and the data were fitted to the Henderson–Hasselbalch equation with a single p*K*_a_ value as follows:

$$\Delta Abs = \frac{v}{{1 + 10^{{\left( {pH - pK_{a} } \right)}} }} + w$$

where *v* represents the amplitude of the change of absorption differences and *w* is the offset. After the measurements, the reversibility of the pH changes of the sample was checked to confirm there was no sample denaturation during the measurements.

Transient time-resolved absorption spectra of the *E. coli* membrane expressing LaSzR2 from 380 to 700 nm at 5 nm intervals were obtained using a homemade computer-controlled flash-photolysis system equipped with an Nd: YAG laser as an actinic light source^15^. By using an optical parametric oscillator, the wavelength of the actinic pulse was tuned at 545 nm. The pulse intensity was adjusted to 2 mJ per pulse. All data were averaged to improve the signal-to-noise ratio (n = 400–4000). All measurements were conducted at 25 °C. The experiments were performed in buffer (50 mM Tris–HCl (pH 7.0)) containing 300 mM NaCl. To investigate proton uptake and release during the photocycle, we used the pH indicator pyranine (final concentration = 100 µM, Tokyo Chemical Industry Co., Ltd., Tokyo, Japan), which has been extensively used to monitor light-induced pH changes in various rhodopsins^3,15,38^. The pH changes in the bulk environment were measured as the absorption changes of pyranine at 450 nm. The absorption changes of pyranine were obtained by subtracting the absorption changes of the sample without pyranine from those of the sample with pyranine. The *E. coli* membrane samples were suspended in an unbuffered solution containing 300 mM NaCl to enhance the pyranine signals. The results of 10,000-traces were averaged to improve the signal-to-noise ratio.

## Supplementary information

Supplementary Figures.
