# Supplementary material for: Lokiarchaeota archaeon schizorhodopsin-2 (LaSzR2) is an inward proton pump displaying a characteristic feature of acid-induced spectral blue-shift
Source: Sci Rep. 2020 Nov 30;10:20857. doi: 10.1038/s41598-020-77936-9 (PMC7704677; doi:10.1038/s41598-020-77936-9)
Supplement: Supplementary file 1 — Supplementary Figures. [file 41598_2020_77936_MOESM1_ESM.docx]

**Supporting Information**

**Lokiarchaeota archaeon Schizorhodopsin-2 (LaSzR2) is an inward proton pump displaying a characteristic feature of acid-induced spectral blue-shift**

Keiichi Kojima^1, #^, Susumu Yoshizawa^2, #^, Masumi Hasegawa^2^, Masaki Nakama^1^, Marie Kurihara^1^, Takashi Kikukawa^3,4^, & Yuki Sudo^1,2,*^

^1^Graduate School of Medicine, Dentistry and Pharmaceutical Sciences, Okayama University, Okayama 700-8530, Japan.

^2^Atmosphere and Ocean Research Institute, The University of Tokyo, Chiba 277-8564, Japan.

^3^Faculty of Advanced Life Science, Hokkaido University, Sapporo 060-0810, Japan

^4^Global Station for Soft Matter, GI-CoRE, Hokkaido University, Sapporo 001-0021, Japan

^#^These authors contributed equally to this work.

*To whom correspondence should be addressed.

Yuki Sudo; Telephone: +81-86-251-7945, E-mail: [sudo@okayama-u.ac.jp](mailto:sudo@okayama-u.ac.jp)


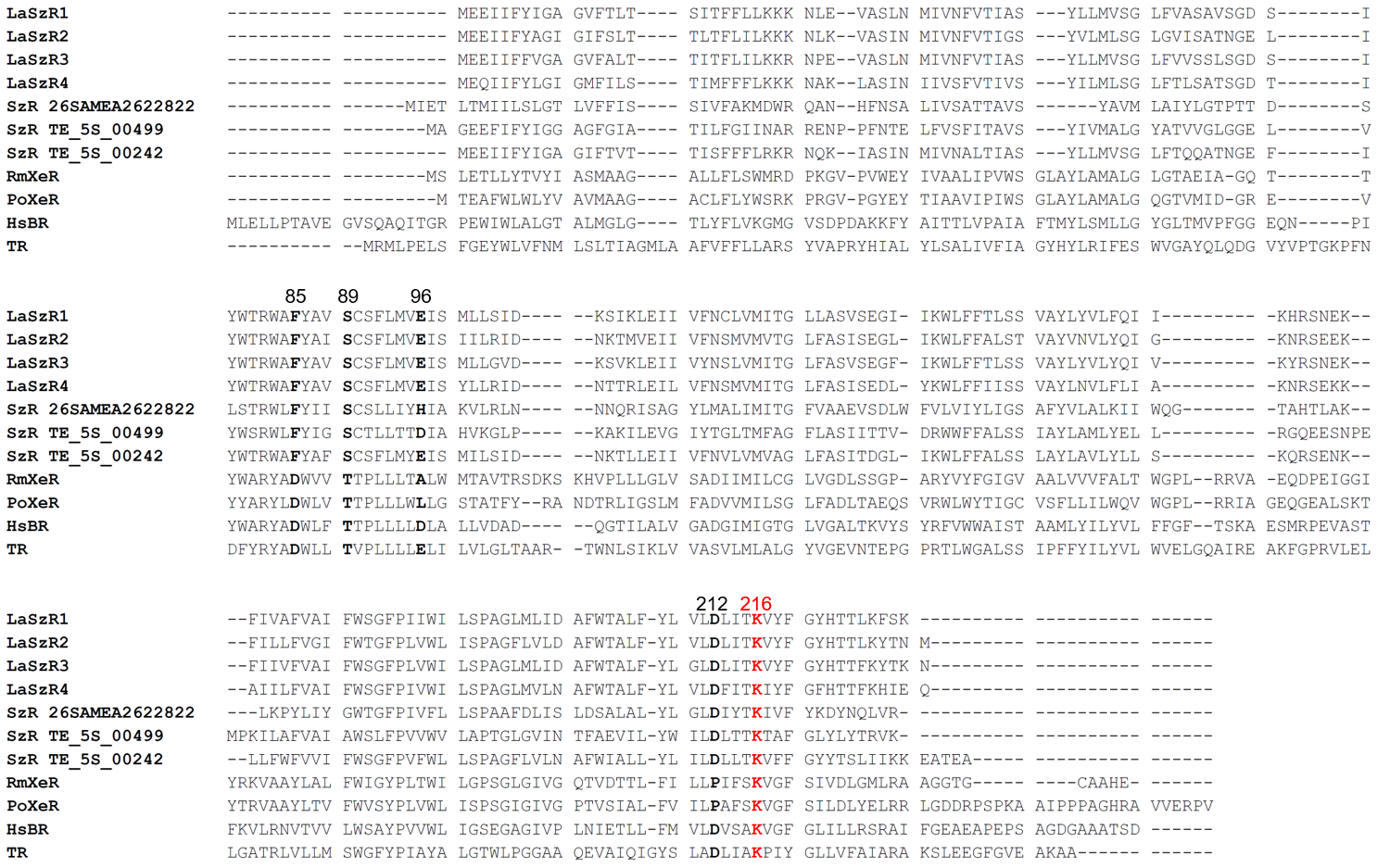


**Figure S1. Amino acid sequence alignment of schizorhodopsins and known microbial rhodopsins.** The accessions of known rhodopsins are follows: LaSzR1(SzR AM_5S_00009, TFG18381), LaSzR2 (SzR un_Tekir_02407, QBQ84358), LaSzR3 (SzR TE_5S 00009, TFG03937), LaSzR4 (SzR AM_5_00977, TFG21677), SzR 26SAMEA2622822 (SzR 26SAMEA2622822_312577, TFH15433), SzR TE_8S_00499 (TFF95899), SzR_TE_8S_00242 (QBQ84355), RmXeR (WP_094549673), PoXeR (WP_051881467), HsBR (CAP14056) and TR (WP_014629850). The critical amino acid residues are shown in bold, and the numbers of amino acids in HsBR are indicated above the columns. The known functions of the amino acids are as follows: primary proton acceptor (Asp85), proton donor (Glu96), proton release group (Glu194 and Glu204), counterion (Asp212), and the Schiff base (Lys216). The Schiff base Lys (K) is shown in red.


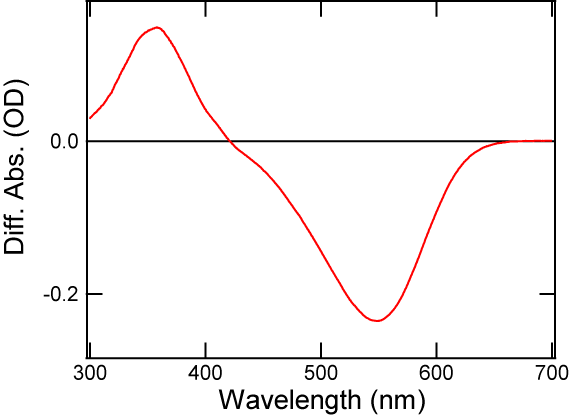


**Figure S2. Difference spectrum of LaSzR2 before and after the incubation with hydroxylamine.** The purified LaSzR2 was incubated with hydroxylamine (final concentration of 100 mM) under light illumination using a xenon lamp (MAX-303, Asahi spectra Co. Ltd., Japan) with a cut-off filter (>420 nm) at room temperature. The difference spectrum after 120 min incubation was shown.


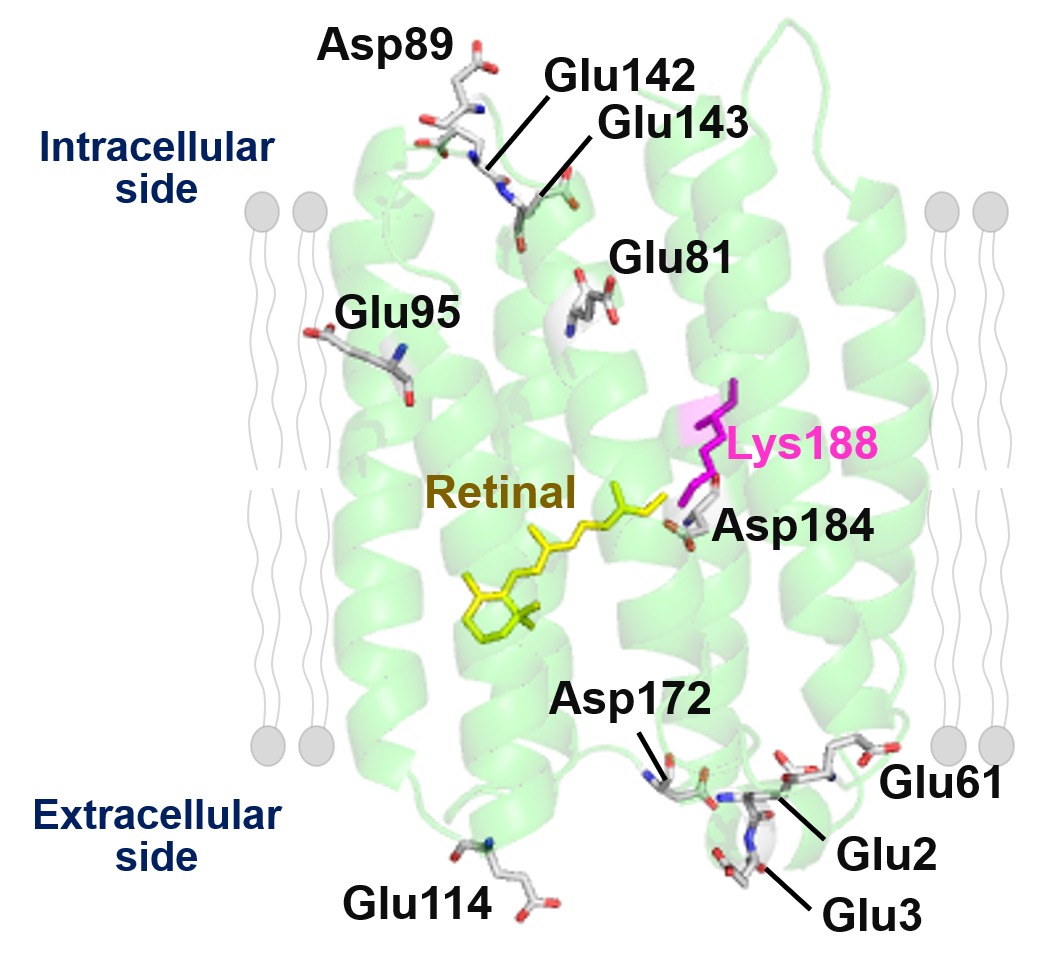


**Figure S3. Model structure of LaSzR2.** The homology model of LaSzR2 was constructed from the crystal structure of *Nanosalina* XeR (PDB 6EYU) by SWISS model (https://swissmodel.expasy.org/). Carboxylates, Lys188 and retinal are coloured gray, magenta, and yellow, respectively.
